# Insights from magnetic resonance imaging of left ventricular non-compaction in adults of North African descent

**DOI:** 10.1186/1755-7682-5-10

**Published:** 2012-03-09

**Authors:** Amal Lachhab, Nawal Doghmi, Youssef Elfakir, Omar Taoussi, Aatef Benyass, Laila Haddour, Jamila Zarzur, Rhizlane Cherradi, Ibtissam Fellat, Aicha Aouad, Fedoua Ellouali, Ilyas Asfalou, Amin Elmajhad, Mohamed Cherti

**Affiliations:** 1 Cardiology B Department, Ibn Sina University Hospital, Rabat, Morocco; 2 Radiology Nakhil Department, Agdal Clinic, Rabat, Morocco; 3 Military Cardiology Department, Ibn Sina University Hospital, Rabat, Morocco

## Abstract

**Background:**

Left ventricular non-compaction (LVNC) is a recently recognized rare disorder. Magnetic resonance imaging (MRI) may help to clarify the uncertainties related to this genetic cardiomyopathy. Despite the fact that many articles have been published concerning the use of MRI in the study of LVNC, there is a lack of data describing the disease in the North African population. The aim of our study is to clarify MRI findings of LVNC in North African patients.

**Methods:**

In our retrospective cohort, twelve patients (7 male, mean age 53 ± 8 years) underwent MRI for suspected LVNC. Correlations were investigated between the number of non-compacted segments per patient and left ventricular ejection fraction (LVEF), then between the number of non-compacted segments and left ventricular end diastolic diameter. The presence or absence of late gadolinium enhancement (LGE) was qualitatively determined for each left ventricular myocardial segment.

**Results:**

Non-compaction was more commonly observed at the apex, the anterior and the lateral walls, especially on their apical and mid-cavity segments. 83% of patients had impaired LVEF. There was no correlation between the number of non-compacted segments per patient and LVEF (r = -0.361; p = 0.263), nor between the number of non-compacted segments per patient and left ventricular end diastolic diameter (r = 0.280; p = 0.377). LGE was observed in 22 left ventricular segments. No association was found between the pattern of fibrosis and non-compaction distribution (OR = 2.2, CI [0.91-5.55], p = 0.076).

**Conclusion:**

The distribution of LVNC in North African patients does not differ from other populations. Ventricular dysfunction is independent from the number of non-compacted segments. Myocardial fibrosis is not limited to non-compacted areas but can extend to compacted segments.

## Background

Left ventricular non-compaction (LVNC) is a rare form of cardiomyopathy characterized by excessive and prominent trabeculations associated with deep recesses that communicate with the ventricular cavity [1,2]. Since its first report in 1984, this disorder has attracted increased attention and still remains the subject of much debate [3].

Magnetic resonance imaging (MRI) is a non-invasive exploration increasingly being employed in the diagnosis of LVNC. Due to its high quality cardiac imaging, this examination method may help to characterize and understand this disease. Thus, several MRI studies have been published describing LVNC features [4-14], yet few of them have focused on the North African population. Accordingly, we decided to report our experiences and describe MRI findings in North African patients, especially the distribution of fibrosis and the contribution of the number of non-compacted segments to left ventricular systolic dysfunction (LVSD).

## Method

### Patient population

We retrospectively reviewed morphological cine magnetic resonance imaging findings of ventricular non-compaction in twelve patients between 2007 and 2010. Those patients had been referred for MRI examination in order to investigate suspected LVNC or dilated cardiomyopathy. Information regarding presenting symptoms and echocardiographic data were obtained from referring centers. Patients with associated coronary artery disease were ruled out of the study.

### CMR

#### CMR acquisition protocol

MRI was performed using a 1.5-Tesla magnetic resonance scanner (Siemens Medical Systems). An eight-element cardiac phased-array receiver surface coil, with breath- holding in expiration and electrocardiogram (ECG) gating, was used for signal reception. The MRI sequences were conducted as follows: echo planar cine true-fisp imaging; dark-blood turbo spin echo T1-weighted; dark-blood turbo spin echo T2-weighted; and dark-blood half-Fourier single shot turbo spin echo. Images were obtained in two-chamber, four-chamber, and short-axis (from the atrioventricular ring to the apex) planes. After baseline imaging, a bolus injection of gadolinium was administered. Ten minutes later, delayed enhancement MRI was performed using an inversion recovery-prepared gated fast gradient-echo pulse sequence. The late gadolinium enhancement (LGE) images were acquired in end-systole in the same views used for cine images.

#### CMR imaging data analysis

All examinations were transferred to a dedicated workstation. Cine images were analyzed using Argus post-processing software (Siemens Medical Systems). The cine loops were reviewed and the end-diastolic and end-systolic frames were identified.

#### LVNC

Assessment of LVNC was performed on short and long axis slices. All left ventricular (LV) myocardial segments were visually screened for the presence of a two-layer structure, with a thin epicardial compacted layer and a thick non-compacted endocardial one, with excessive trabeculations and deep intertrabecular recesses that communicate with the ventricular cavity. The ratio of non-compacted to compacted (NC/C) myocardium was measured for each involved myocardial segment in diastole, on short axis slices. For each patient, we considered the minimal NC/C ratio for subsequent analysis. Non-compaction was defined as a ratio of NC/C myocardium > 2.3 at end-diastole [6]. The distribution of non-compaction was assessed by qualitative analysis of all seventeen segments. Segmental analysis was evaluated using a standard seventeen-segment cardiac model as defined by the American Heart Association/American College of Cardiology (AHA/ACC) for standardized myocardial segmentation [15].

#### LV diameters, volumes, and ejection fraction

LV diameters, volumes and ejection fraction (EF) were measured using standard volumetric techniques with dedicated software [16]. Left ventricular systolic dysfunction was defined as a left ventricular ejection fraction (LVEF) < 50%.

For each patient, correlations were investigated between the number of non-compacted segments and EF, then between the number of non-compacted segments and LV end diastolic diameter.

#### Late gadolinium enhancement

The presence of late gadolinium enhancement (LGE), a surrogate of myocardial fibrosis, was qualitatively determined for each LV myocardial segment using the seventeen-segment cardiac model. All short and long axis contrasted-enhanced images were reviewed, with a particular focus on images with elevated signal intensity. Patterns of LGE were visually classified as sub-endocardial, sub-epicardial, mid-myocardial, or transmural (LGE occupying ≥ 75% of LV wall thickness).

### Statistical analysis

The data was assembled using the computer software "EXCEL" (Microsoft). All analyses were performed with SPSS (version 18.0), in collaboration with the Faculty of Medicine's Biostatistics Department at the University of Rabat. Continuous variables are expressed as the mean and the standard deviation, and nominal variables as numbers and percentages. Univariate and multivariate analyses were made with the binary logistic regression model. Pearson correlation analyses were used for univariate correlations. A two-tailed p value of < 0.05 was considered statistically significant.

Because this study was done retrospectively, no ethics committee approval was required.

## Results

### Baseline characteristics

A total of twelve (n = 12) cases of LVNC were included in our study. The mean age was 53 ± 8 years. Seven (58%) patients were male; two 2 (17%) were of sub-Saharan descent. Most patients (92%) were symptomatic. Six patients presented with dyspnea and four with palpitations. One patient had both dyspnea and chest pain. In seven patients, LVNC had been previously suspected on echocardiography. Four cases had been labeled as dilated cardiomyopathy. One case has the diagnosis of LVNC been made by echocardiography. (Table 1)

**Table 1:** Baseline characteristics of study population

Patient	Age (years)	Sex	Race	symptom	TTE
patient 1	56	M	white	palpitations	suspected LVNC
patient 2	56	M	white	dyspnea	LVNC
patient 3	45	M	white	dyspnea	suspected LVNC
patient 4	68	F	white	dyspnea	suspected LVNC
patient 5	58	F	white	dyspnea dyspnea, chest	suspected LVNC
patient 6	56	F	white	pain	suspected LVNC biventricular dilated
patient 7	51	M	black	palpitations	cardiomyopathy
patient 8	50	F	white	asymptomatic	dilated cardiomyopathy
patient 9	52	M	black	dyspnea	dilated cardiomyopathy
patient 10	38	M	white	dyspnea	dilated cardiomyopathy
patient 11	49	F	white	palpitations	suspected LVNC
patient 12	60	M	white	palpitations	suspected LVNC

M: male gender; F: Female gender; LVNC: Left ventricular non compaction; TTE: transthoracic echocardiography;

### CMR imaging findings

The mean LV end-diastolic diameter and the mean LVEF were 66 ± 11 mm and 35% ± 16%, respectively. A total of ten (83%) patients had impaired LVEF (LVEF < 50%). Among the segments analyzed in our study, 120 (59%) were identified as non-compacted segments. The mean number of non-compacted segments per patient was 10 ± 2.7 and the mean NC/C ratio was 2.8 ± 0.5. (Table 2)

**Table 2:** Cardiac magnetic resonance imaging characteristics of study population

Patient	LVEF (%)	LVD (mm)	NC/C	number of NC segments	NC segments	LGE
patient 1	35	53	2,3	10	Apex Apical segments anteroseptal, anterior and antero-lateral mid-cavity segments anterior and antero-lateral basal segments right ventricular	_

patient 2	20	86	2,5	9	.apex antero-lateral wall infero-lateral wall inferior wall	_

patient 3	32	70	3	12	all segments except the septum wall	Sub-endocardial on the antéro-septal wall

patient 4	28	64	2,3	12	all segments except the septum wall	_

patient 5	20	68	2,3	7	apex anterior, lateral and infero- apical segments anterior, antero-lateral and inferolateral mid-cavity segments	Midmyocardial on the antero-septal wall

patient 6	38	58	2,5	9	apex apical segments anterior, antero-lateral, inférolatérale and inferior mid- cavity segments	_

patient 7	20	77	2,6	12	apex apical segments anterior, anterolateral, inferolateral and inferior mid-cavity segments anterior, anterolateral and inferolateral basal segments right ventricular	_

patient 8	38	59	3,6	14	apex anterior, lateral and infero apical segments anteroseptal, anterior, anterolateral, inferolateral and inferior mid-cavity segments anteroseptal, anterior, anterolateral, inferolateral and inferior basal segments	Midmyocardial on the infero-septal wall

patient 9	10	82	2,4	12	all segments except the septum wall right ventricular	Sub-endocardial involving the apex, the apical and mid-cavity segments, and the septal and lateral basal segments

patient 10	47	63	3,2	11	apex anterior, lateral and infero apical segments anteroseptal, anterior, anterolateral, inferolateral and inferior mid-cavity segments anterolateral and inferolateral basal segments	_

patient 11	56	56	3,9	5	anterior, lateral and infero apical segments Anterolateral and inferolateral mid-cavity segments	_

patient 12	50	56	2,5	7	apex anterior, lateral and infero apical segments anterolateral, inferolateral and inferior mid-cavity segments	_

**Figure 1:**
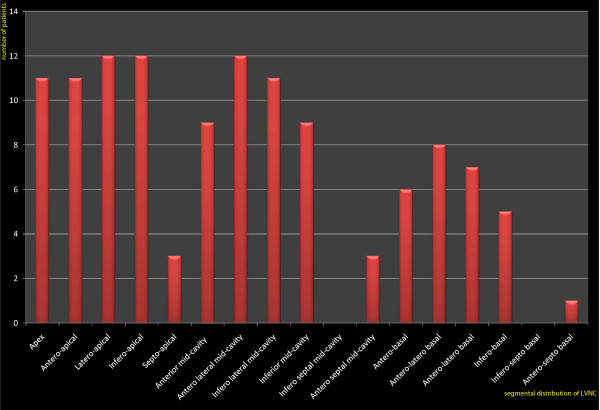
**Distribution of non compacted myocardium according to LV segments**. Non-compaction is more commonly observed at the apex, the anterior wall and the lateral wall, especially on their apical and mid-cavity segments.

**Figure 2:**
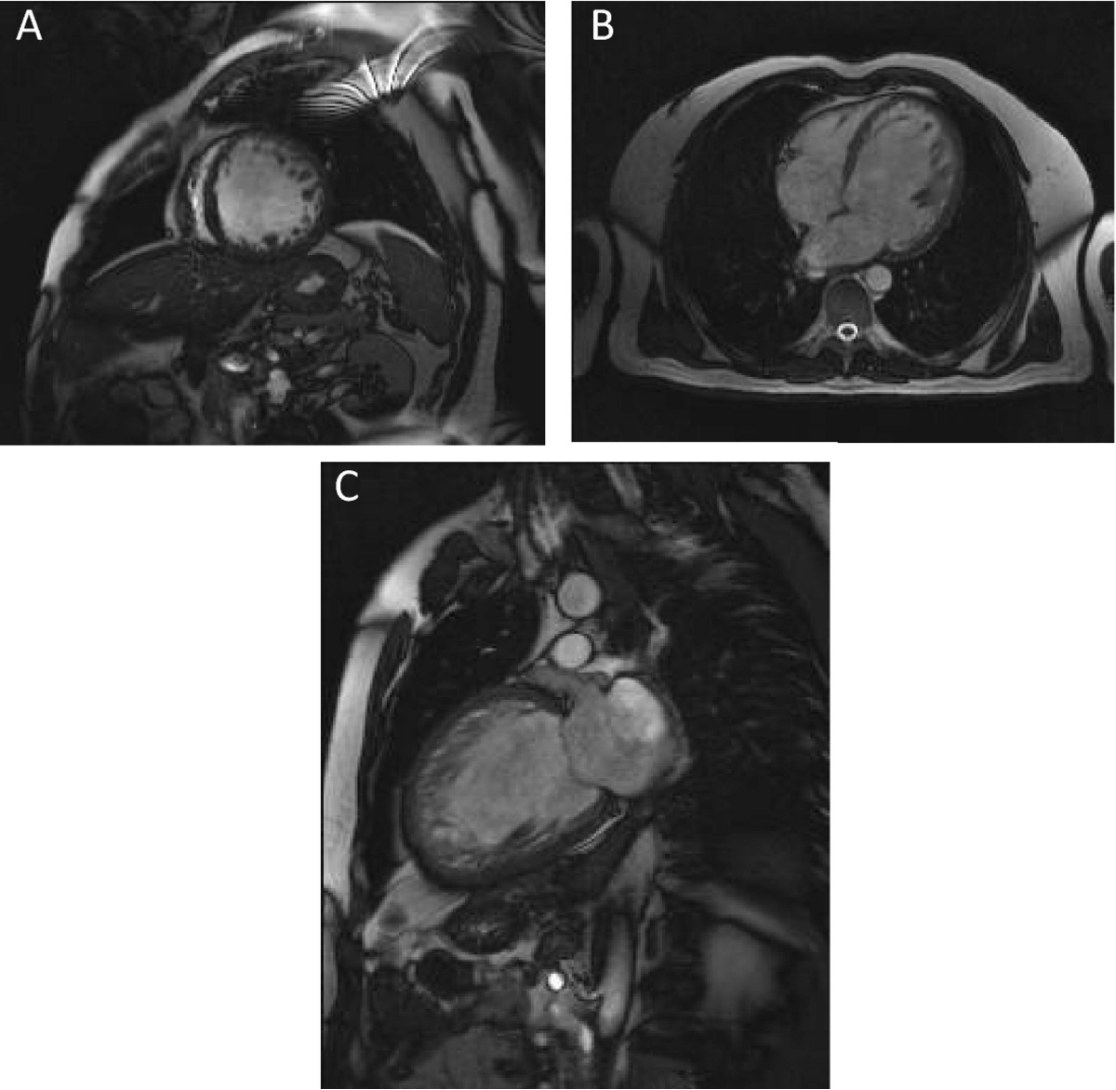
**MR appearences of LVNC showing two layer structure with prominent trabeculations and deep intertrabecular recesses**. A: short axis image; B: four chamber image; C two chamber image. CMR images of patients with LVNC.

NC/C: non compacted/compacted ratio; LVD: left ventricular end diastolic diameter; LGE: late gadolinium enhancement; LVEF: left ventricular ejection fraction;

As shown in Figure 1, non-compaction was more commonly observed at the apex, the anterior wall and the lateral wall, especially on their apical and mid-cavity segments. The involvement of basal segments was less frequent. Infero-septal wall involvement was not found in any patient. In our series, right ventricular involvement has been noted in three (25%) cases. Figure 2 shows examples of CMR images of patients with LVNC.

**Figure 3:**
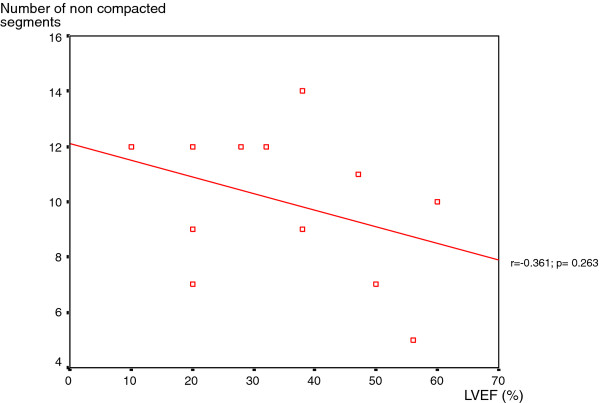
**Relation between the number of no compacted segments per patient and the left ventricular ejection fraction**. There is no correlation emerged between the number of non-compacted segments per patient and systolic dysfunction.

**Figure 4:**
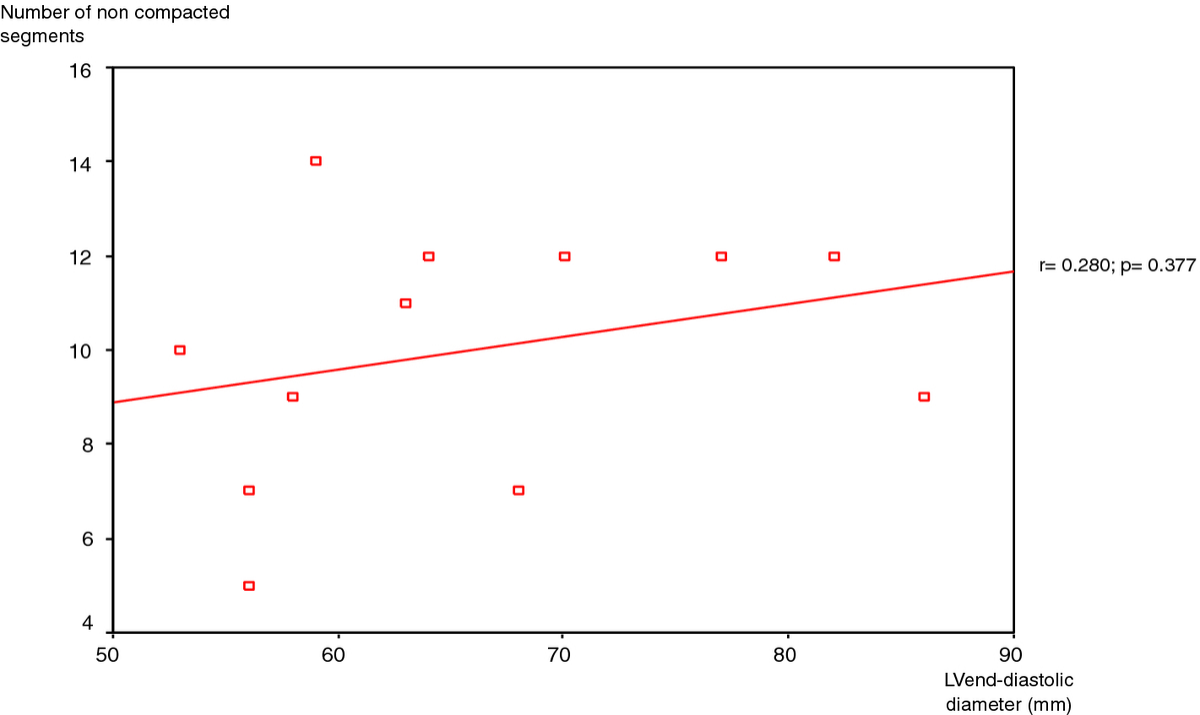
**Relation between the number of no compacted segments per patient and the left ventricular end diastolic diameter**. There is no correlation between the number of non-compacted segments per patient and LV end diastolic diameter.

In analyses of the study group, no correlation emerged between the number of non-compacted segments per patient and systolic dysfunction (r =-0.361; p = 0.263), nor between the number of non-compacted segments per patient and LV end diastolic diameter (r = 0.280; p = 0.377) [Figures 3, 4].

**Figure 5:**
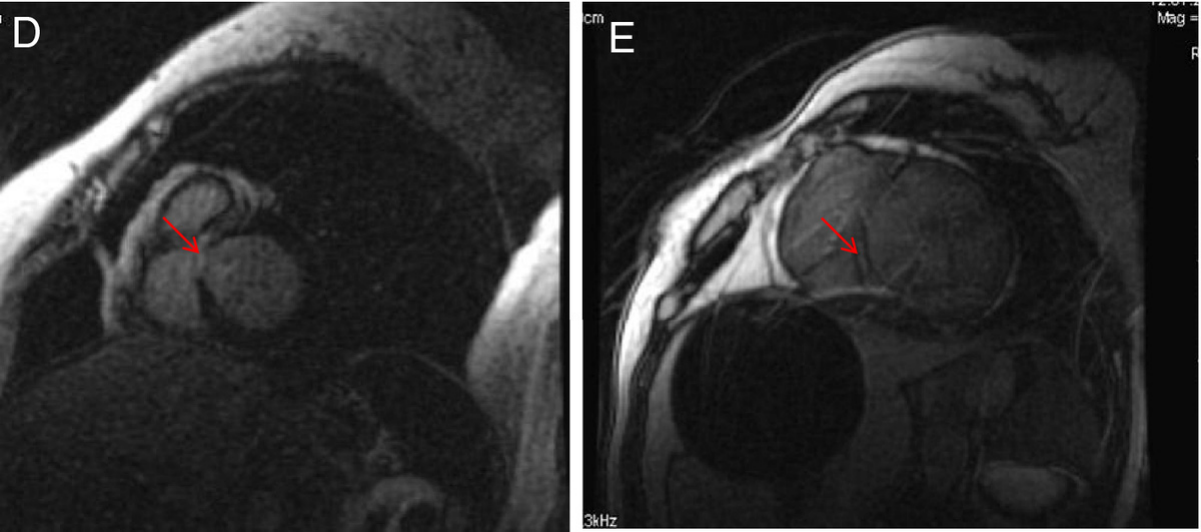
**Short axis delayed contrast images showing late gadolinium enhancement (fibrosis)**. D: sub-endocardial LGE; E: mid- myocardial LGE. LGE: late gadolinium enhancement. CMR images of late gadolinium enhancement.

**Figure 6:**
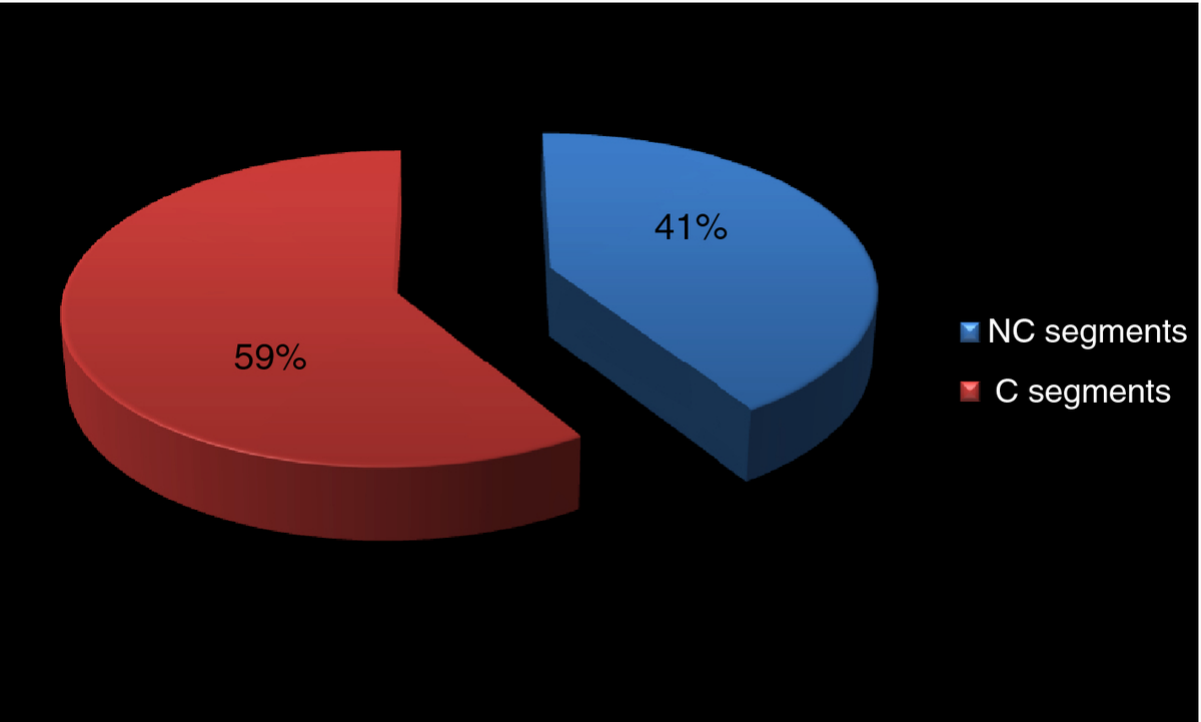
**Distribution of LGE segments**. C: compacted; NC: non compacted; LGE: late gadolinium enhancement. LGE was seen in twenty-two LV segments, thirteen (59%) of which were compacted segments, the others (41%) were non-compacted.

**Figure 7:**
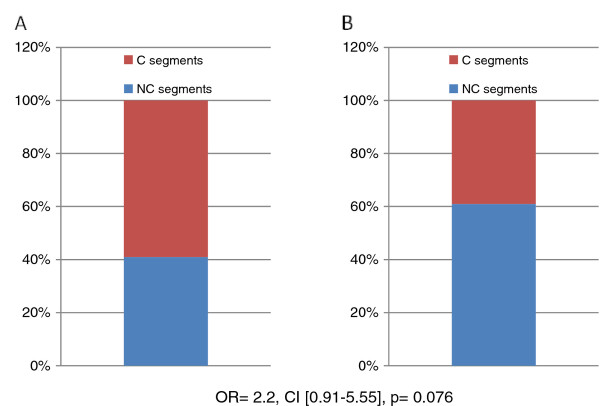
**Association between the patern of LGE segments and NC distribution**. A: segments with LGE; B: segments without LGE. C: compacted; LGE: late gadolinium enhancement; NC: non compacted. NC segments: non compacted segments; C segments: compacted segments; LGE: late gadolinium enhancement. There is no association between the pattern of fibrosis and non-compaction distribution.

A total of four (33%) patients showed LGE. LGE was mid-myocardial in two cases and sub-endocardial in the remaining patients [Figure 5]. Overall, LGE was seen in twenty-two LV segments, thirteen (59%) of which were compacted segments, while the others (41%) were non-compacted [Figure 6]. There was no *significant discrepancy *between non-compacted and compacted segments concerning fibrosis involvement (OR = 2.2, CI [0.91-5.55], p = 0.076) [Figure 7].

## Discussion

The non-compaction of the LV myocardium is a congenital heart disease caused by an arrest of normal ventricular embryogenesis, which causes an excessive number of endomyocardial trabeculae, spaced out by deep recesses [17,18]. Recently, LVNC was classified by the American Heart Association as a genetic cardiomyopathy [19]. Despite the publishing of many papers concerning LVNC, there are still important uncertainties related to this enigmatic disease.

Our study is one of the first series assessing MRI features of LVNC in the North African population. In only one instance was the diagnosis of LVNC confirmed by transthoracic echocardiography. In the others, the diagnosis has been given by MRI. Our findings are in agreement with prior research which asserts that even though LVNC diagnosis remains difficult [20], the use of MRI is superior to transthoracic echocardiography in the detection of non-compaction [4,6,8,12]. Some authors have emphasized the tendency to misdiagnose the appearance of non-compaction on transthoracic echocardiography as concentric hypertrophic cardiomyopathy [21,22], dilated cardiomyopathy or an apical tumor [12,23]. In addition, echocardiography poses inherent problems in both the use on patients with poor acoustic windows as well as the assessment of the left ventricular apex, known to be the most commonly non-compacted area [6]. At present, MRI is being increasingly employed in the diagnosis of LVNC because it has several advantages, such as high spatial resolution, large field of view, multiplanar capability and the ability to detect myocardial scarring [7,24].

The findings of our study are in agreement with those of previous reports: non-compacted segments are most commonly located at the apex and the lateral wall; the septal segments are rarely affected by LVNC [6,25]. It is commonly held that the normal compaction process of LV myocardium goes from the base to the apex and from the septal to the lateral wall [26]. We agree with Peterson et al who stipulate that varying degrees of arrest in this normal embryologic process may provide an explanation for the typical pattern of distribution of non-compacted segments [6].

In our study, we tried to investigate the relationship between the extent of LVNC and LV impairment. Although 83% of patients had impaired LVEF, our analysis of the study group found no correlation between the number of non-compacted segments and LVEF. Previous reports have studied the contribution of non-compacted segments to LV dysfunction. Aras et al found that the number of LV segments affected by non-compaction is a major determinant of LV systolic dysfunction [27]. Lofiego et al were able to positively correlate the number of non-compacted segments with LVEF [28]. In a more recent study, Belanger et al demonstrated that increasing degrees of non-compaction correlate with the worsening of LV systolic function [29]. In the study group of Youssef et al, in which MRI was employed to assess the extent of non-compaction, an inverse correlation between non-compacted areas and LVEF was only noted in patients without dyspnea. Considering the whole population, no correlation emerged between these two parameters [12]. In a larger population, Fazio et al underlined that there is no association between affected segments and LV systolic dysfunction [18]. These findings have been confirmed by Habib et all who found, in the analysis of the non-compaction French registry, that LV dysfunction was not related to the extent of the non-compacted area [20]. Dissimilarities between these reports can be explained by the differences in patient recruitment, the use of different imaging modalities to diagnose the disorder (transthoracic echocardiography or MRI), the clinical stage of disease in the study group, and also the ethnic origin of the considered population. In our study, as previously reported, we used MRI to assess LVNC features in our North African population. As demonstrated, cardiac MRI provides a more accurate and reliable evaluation of the extent of non-compacted myocardium than transthoracic echocardiography [8,9].

A distinct advantage of MRI is its capability of myocardial tissue characterization and its ability to demonstrate myocardial fibrosis with enhanced delayed imaging. Fibrosis is commonly found in association with LVNC, the diagnosis of which is unattainable with other non-invasive imaging modalities [5-7]. In our study, cardiac MRI revealed myocardial fibrosis in four cases. LGE was mid-myocardial in two patients and sub-endocardial in the others. This data is consistent with others' observations in different patient populations [5-10] which confirmed the presence of intra-myocardial and sub-endocardial fibrosis in patient with LVNC.

In our patients, myocardial fibrosis was detected in not only involved segments but also morphologically normal ones, with a similar prevalence in both non-compacted and compacted zones. This result supports the hypothesis that non-compaction may be a diffuse disease process involving both non-compacted and compacted area [10,30]. The patho-physiological mechanisms leading to myocardial fibrosis in isolated LVNC are poorly understood. Jenni et al have demonstrated a diminution in coronary flow reserve in both non-compacted and compacted segments of myocardium in LVNC [31]. Thus, defects in the coronary microcirculation might be responsible for ischemic lesions and myocardial fibrosis.

### Study limitations

The major limitations of our study are its retrospective design and its small sample size, which can be explained by the high cost of MRI. Moreover, patients included in the study were referred for MRI from other centers. Thus, there was a selection bias in this population representing a group of mainly symptomatic patients, so the true severity of the disease was probably overestimated. In addition, pediatric cases were not included. Furthermore, we did not undertake family screening, nor were clinical data and follow up information available. Finally, we were unable to compare echocardiographic and MRI findings because echocardiographic examinations were not performed in the same institution.

## Conclusion

LVNC is an enigmatic cardiomyopathy that is still not widely known. Our study supports the hypothesis that LVNC diagnosis is not always clear and that MRI, with its high spatial resolution, seems to be able to improve the detection of this condition. Although our data refers to a small population, it suggests that the distribution of LVNC in North African patients does not differ from other populations. In addition, the extent of non-compaction appears to be unrelated to LV systolic dysfunction in this population. Furthermore, our findings indicate that myocardial scarring, which can be identified non-invasively by MRI, is not limited to the non-compacted myocardium, but can extend to morphologically normal areas. Finally, further studies of larger populations, including long-term follow up, are still needed in order to better clarify different facets of this cardiomyopathy. Accordingly, we propose the creation of a LVNC registry for North Africa.

## Abbreviations

C: Compacted; CMR: Cardiac magnetic resonance; F: Female gender; LGE: Late gadolinium enhancement; LV: Left ventricular; LVD: Left ventricular end diastolic diameter; LVEF: Left ventricular ejection fraction; LVNC: Left ventricular non-compaction; LVSD: Left ventricular systolic dysfunction; M: Male gender; MRI: Magnetic resonance imaging; NC: Non compacted; TTE: Transthoracic echocardiography.

## Competing interests

The authors declare that they have no competing interests.

## Authors' contributions

The contributions of each author to this manuscript are as follows: AL and ND were involved in the design of the study, in cardiovascular magnetic resonance imaging and analyzing images, in acquisition, analysis and interpretation of the data, and in drafting of the manuscript. YE and OT were involved in cardiovascular magnetic resonance imaging. AB, LH, JZ, RC, IF, AA and IA were involved in echocardiography's examination of the patients. FE and AE were involved in analysis and interpretation of the data. MC was involved in the design of the study and in revising the manuscript. All authors have read and approved the final manuscript.
